# Umbilical Hernia Containing Appendicitis

**DOI:** 10.7759/cureus.8075

**Published:** 2020-05-12

**Authors:** Kevin Sigley, Thomas Russo, Stephen Welch

**Affiliations:** 1 General Surgery, Beaumont Health, Dearborn, USA; 2 Surgery, Beaumont Health, Trenton, USA; 3 Surgery, Beaumont Health, Dearborn, USA

**Keywords:** umbilical hernia, appendix, appendicitis

## Abstract

Umbilical hernia is a common cause for patient presentation to the surgeon, often on a nonemergent basis for a bulge at or lateral to the umbilicus but occasionally under emergency circumstances for pain or bowel obstruction when the hernia contents become incarcerated or strangulated. Risk factors for umbilical hernia include female gender, obesity, and ascites. A defect in the abdominal wall fascia at the umbilicus allows the preperitoneal adipose tissue, omentum, or small or large bowel to protrude through the defect. Rarely described is herniation of the appendix through an umbilical hernia, though appendix-containing femoral hernia (de Garengeot hernia) and appendix-containing inguinal hernia (Amyand hernia) are more common. There are 10 available case reports in the medical literature that describe an appendix-containing umbilical hernia; in this case report, we present the 11th case report of appendicitis within an umbilical hernia.

## Introduction

Umbilical hernia is very common in the United States, with an estimated prevalence of 23% to 50% of the population, with a 3:1 female-to-male distribution [1]. There were an estimated 567,000 emergent hernia operations performed in the United States between 2001 and 2010 [2]. The contents of umbilical hernias are most commonly fat, omentum, and small bowel, though occasionally they may contain other organs [3,4]. Appendix-containing femoral and inguinal hernias possess eponyms (de Garengeot and Amyand, respectively); however, the rarity of umbilical hernia containing appendix possesses no eponym as of yet. There are 10 available case reports describing umbilical hernia containing appendicitis; herein we present the 11th case report of appendicitis within an umbilical hernia.

## Case presentation

A 57-year-old male presented to the emergency department complaining of a mass at his umbilicus with pain in the mass. He reported developing an umbilical hernia seven months prior to admission but that he was able to reduce the hernia with direct pressure. Four days prior to the day of admission, the patient developed worsening pain at the hernia and was unable to reduce the hernia. He denied any other symptoms including fever, nausea, vomiting, diarrhea, constipation, and urinary changes. The patient had no prior surgical history, took no medications, worked as a bartender, and drank alcohol socially. There was no pertinent family history.

**Figure 1:**
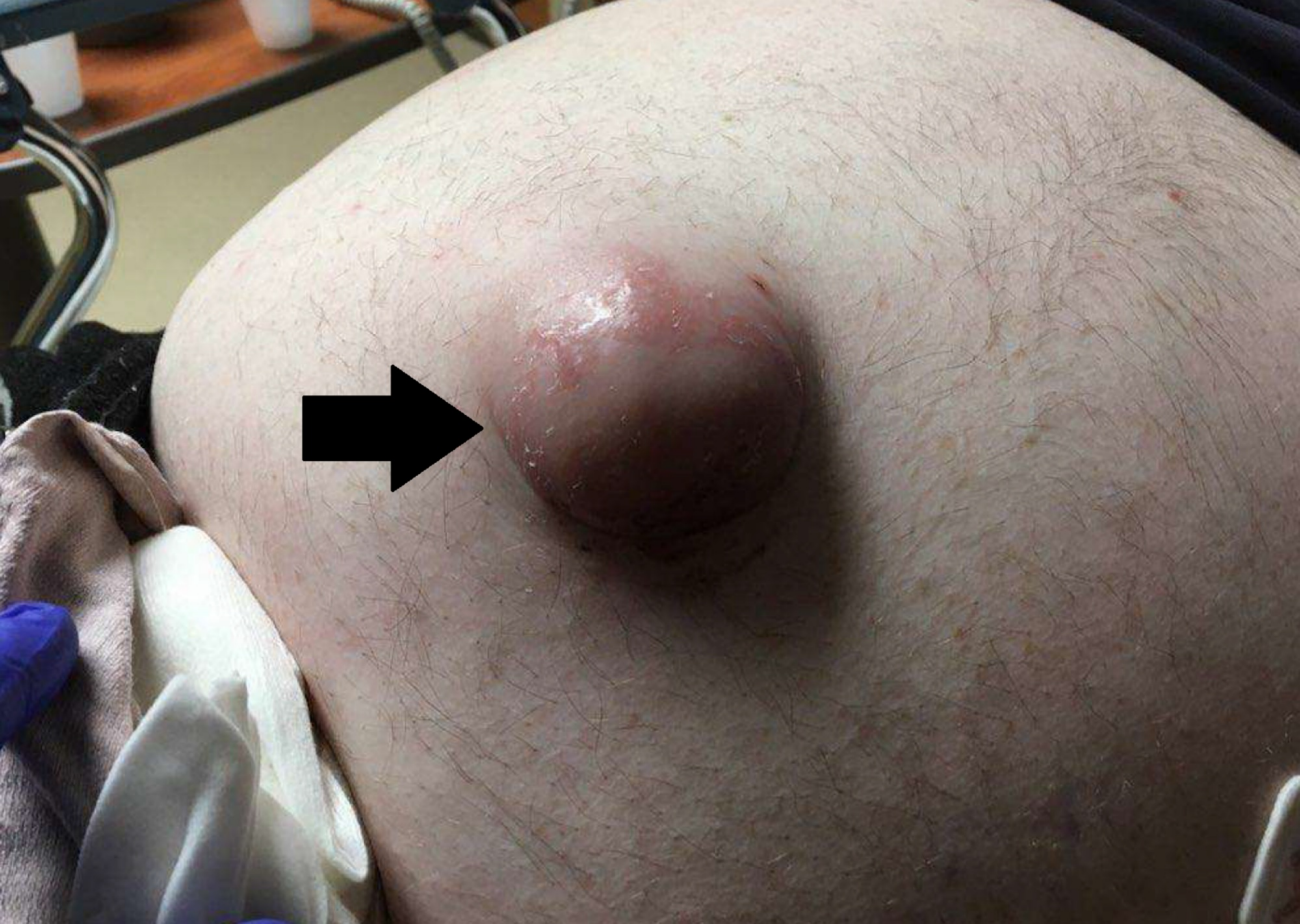
Photo of umbilical hernia on presentation showing overlying skin changes.

Physical examination revealed an obese middle-aged male in mild pain distress, with blood pressure of 148/86, pulse of 80 beats/minute, oral temperature of 99.2°F (37.3 °C), respiratory rate of 20 breaths/minute, SpO_2_ of 94%, and a BMI of 37.59 kg/m². The abdomen was obese and soft, with a large, firm, and tender umbilical hernia, which obscured the umbilicus, with overlying skin changes of erythema and ecchymosis (Figure 1). Auscultation of the heart and lungs revealed a regular rate and rhythm without murmur, and clear breath sounds.

**Figure 2:**
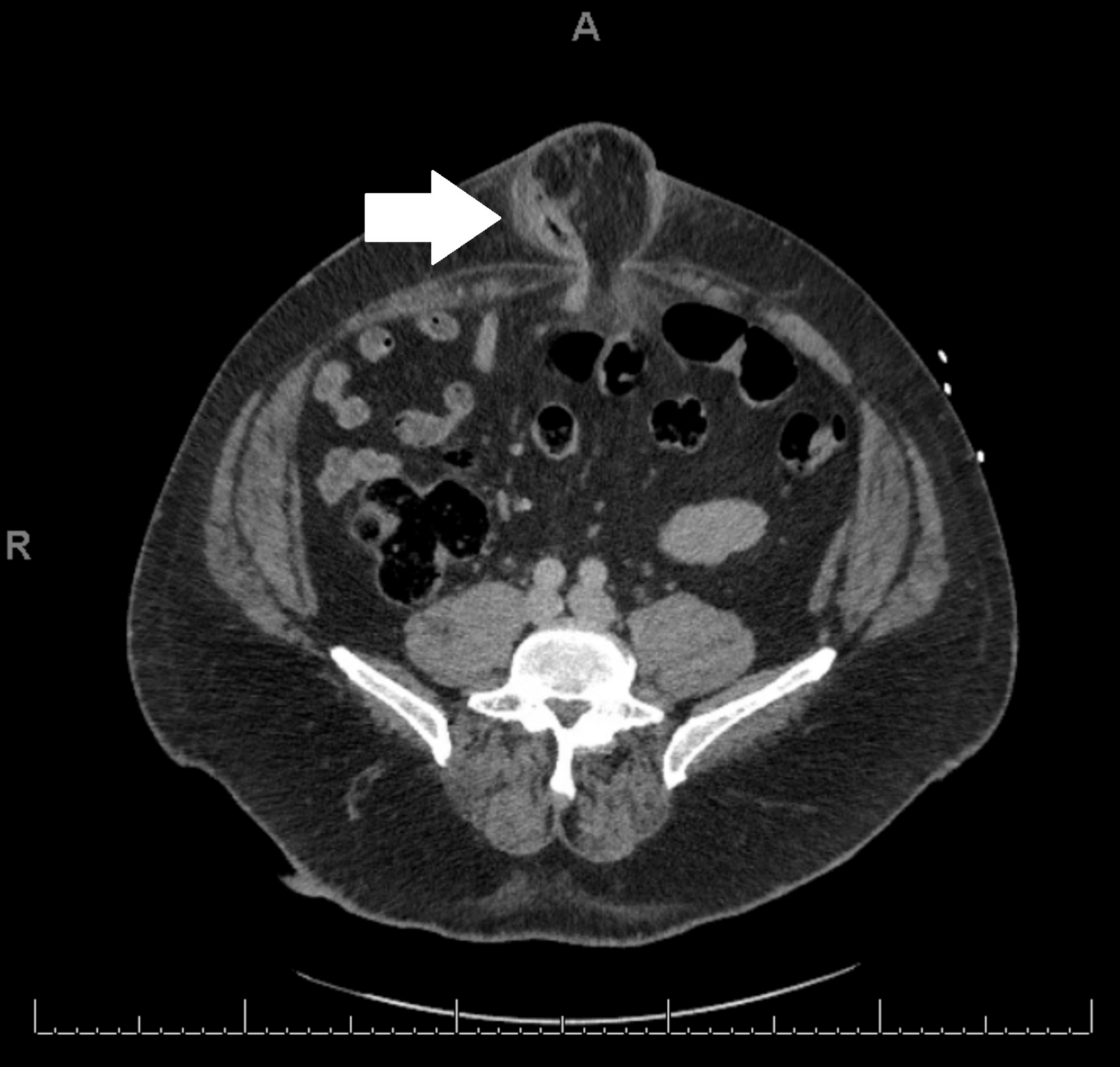
Axial CT showing umbilical hernia containing the appendix.

**Figure 3:**
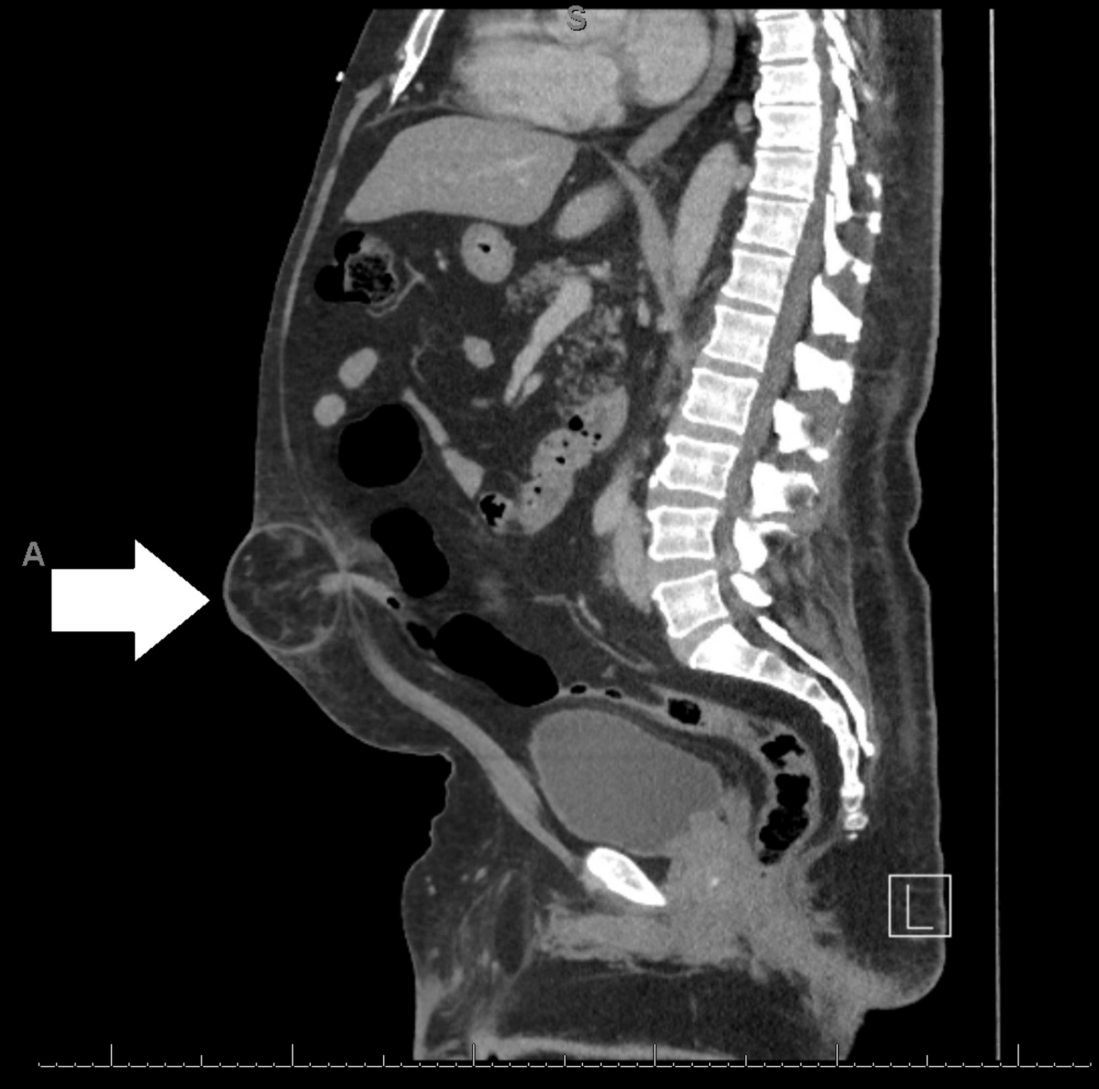
Sagittal CT showing umbilical hernia containing the appendix.

CT imaging and laboratory evaluation were obtained, which revealed a large umbilical hernia, with suspected strangulated small bowel with pneumatosis intestinalis (Figures 2, 3). However, there was no evidence of bowel obstruction, and though the patient was tender at the umbilical hernia, the degree of pain was less than would be expected for strangulated bowel. Laboratory evaluation was within normal limits, with no leukocytosis or lactic acidosis.

**Figure 4:**
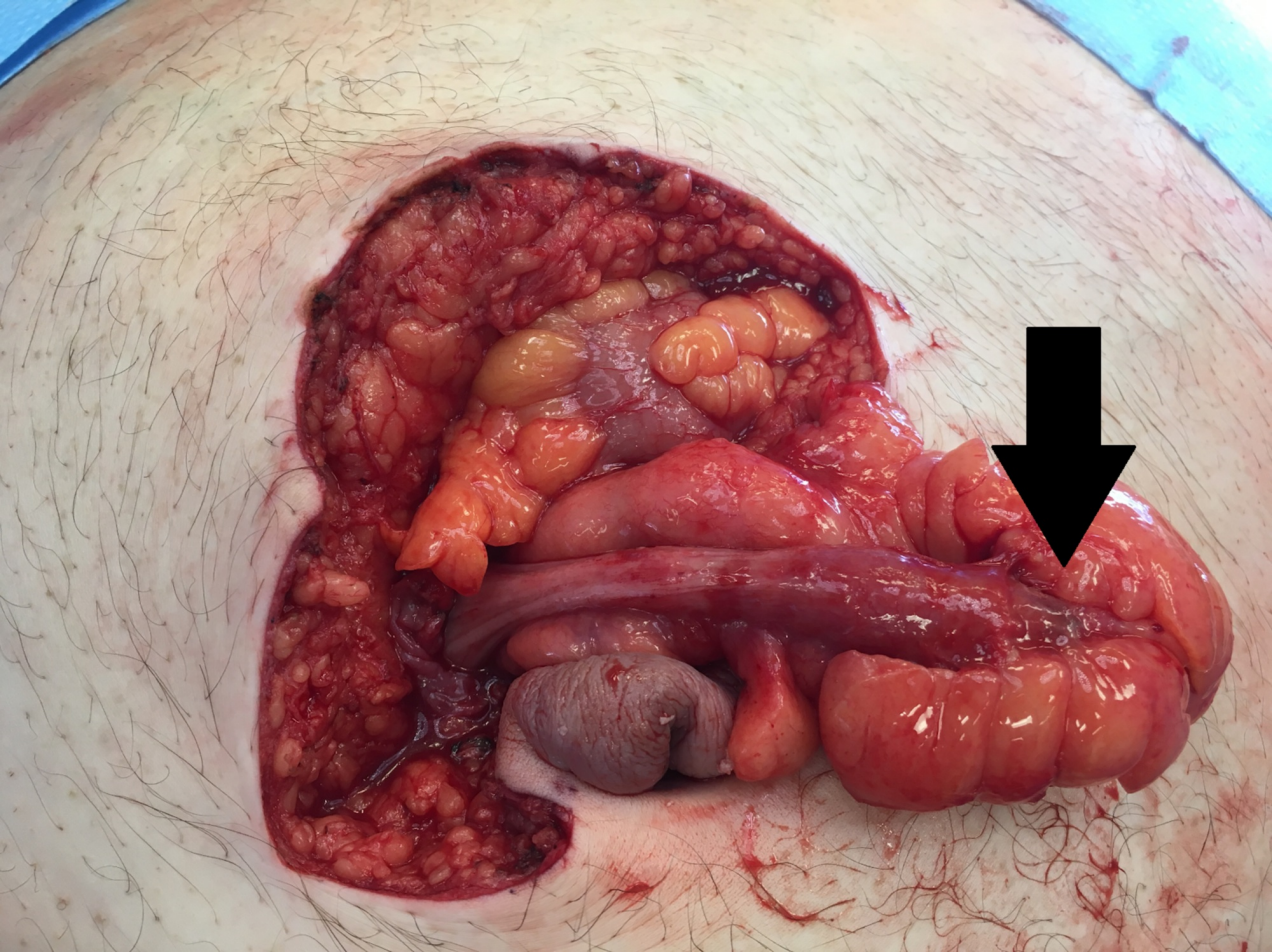
Intraoperative photo showing the appendix and mesoappendix dissected from the hernia sac.

Given the CT findings in conjunction with the patient’s pain, the patient was taken urgently to the operating room due to concern for strangulated small bowel in the umbilical hernia. Exploratory laparotomy was undertaken, with the incision initiated superior to the mass and extended to the patient’s right around the hernia and then inferior to the hernia. The dissection to the fascia was undertaken with a no. 10 blade and blunt dissection to prevent thermal injury to underlying structures due to the thin hernia sac. The hernia sac was dissected free, incised, and explored. A tubular blind-ended structure was encountered (Figure 4), which was determined to be the vermiform appendix, with evidence of necrosis at the tip. Open appendectomy was performed by creating a window at the base of the mesoappendix, using a 55 GIATM stapler (Medtronic, Minneapolis, MN) for the appendix and silk tie for the mesoappendix. The hernia sac also contained a small piece of necrotic appearing omentum, which was suture-ligated and removed.

The appendix, omentum, and hernia sac were sent to pathology, which later reported the appendix to show changes consistent with acute appendicitis. The excised portion of the omentum revealed fat necrosis, and focal acute and chronic inflammation.

**Figure 5:**
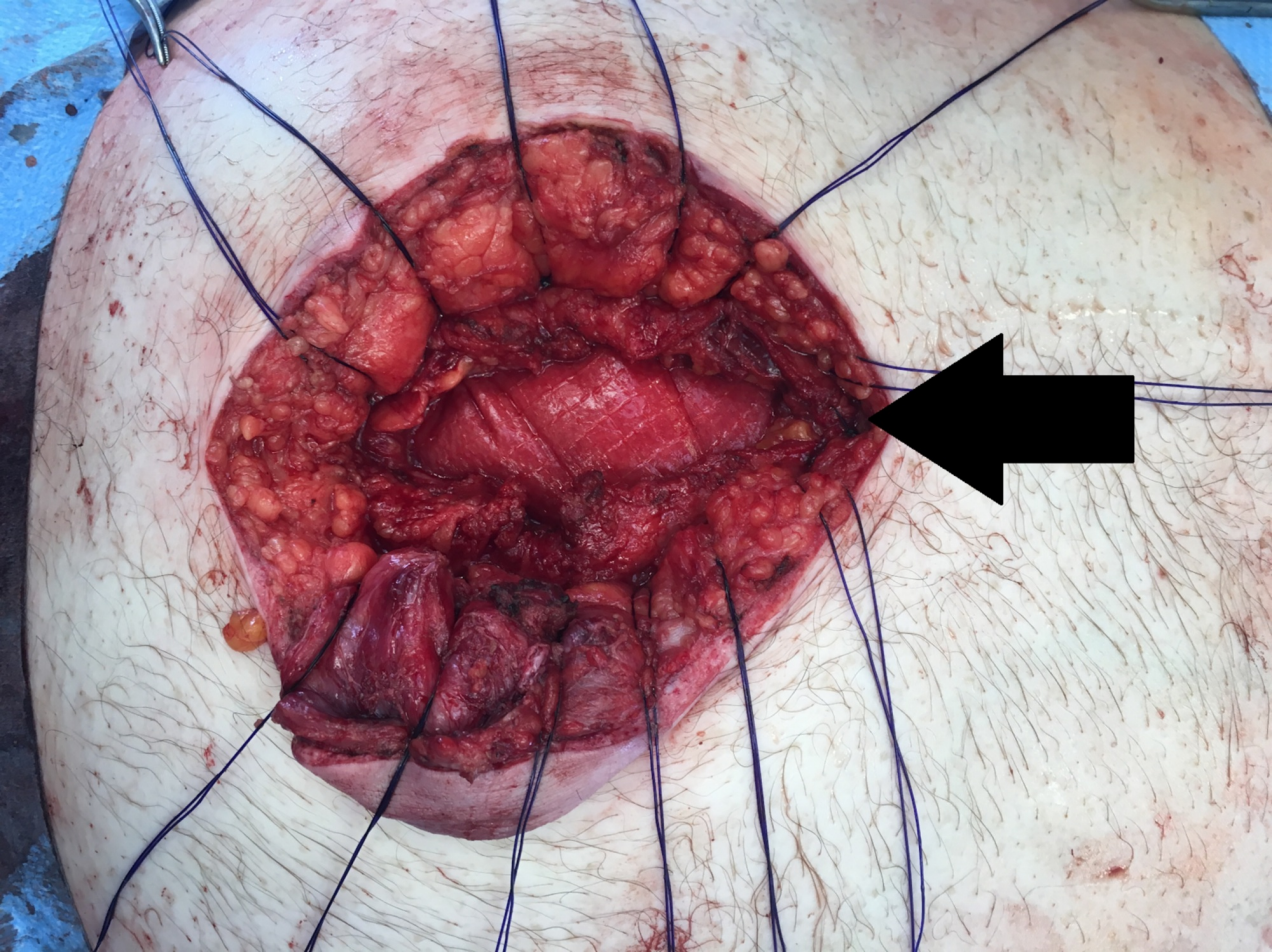
Intraoperative photo showing 12 x 20 cm resorbable mesh repair of the umbilical hernia defect.

The small bowel was examined and found to be normal in appearance. The resultant fascial defect was 6 cm x 9 cm, and a 12 cm x 20 cm underlay bioscaffolding resorbable mesh was placed and secured with 0 PDS® suture (Ethicon, Somerville, NJ, USA) (Figure 5). Given the concern for infection in the field, a biologic mesh was selected in favor of a synthetic mesh. The fascia was closed using figure-of-eight 0 Vicryl® sutures (Ethicon) followed by 0 PDS® sutures in a simple interrupted fashion. The subcutaneous tissues were then closed using 3-0 Vicryl® sutures in a simple interrupted fashion. The skin was closed using skin staples. The patient did well in the postoperative period and was discharged home on postoperative day 3.

## Discussion

The usual process of development of appendicitis is obstruction of the appendiceal lumen and increase in intraluminal and intramural pressure, which leads to small vessel occlusion and lymphatic stasis, distension of the appendix, and appendiceal wall ischemia and necrosis [5]. Whether the location of the appendix within the hernia contents played a role in the development of acute appendicitis in this case is not known, but given the presence of omental necrosis, strangulation of the hernia contents or compression of the appendix at the hernia neck likely resulted in the development of acute appendicitis. There was no evidence of abscess or perforation.

Although the base of the appendix is reliably found at the cecum, the course and location of the appendix are variable, with the most common location being retrocecal [5]. In this case, the original course of the appendix likely was preileal and directed medially from the base of the cecum. The exact mechanism of the appendix protruding through the umbilical defect and becoming incarcerated is not known, though it is posited that it may be due to mobile cecum or cecal bascule. The standard of care for appendicitis without perforation is appendectomy. In this case, at the time of operation, the appendix appeared necrotic, and as there was no evidence of abscess or perforation, the decision was made to repair the hernia concurrently with a biologic mesh as opposed to a synthetic one.

Appendicitis presenting in an incarcerated umbilical hernia is an extremely rare phenomenon, with only 10 case reports of similar nature being available in the medical literature [6-8]. In this case, the suspected umbilical hernia contents were the omentum and small bowel, though there was no evidence of small bowel obstruction, which would be expected with a strangulated umbilical hernia containing small bowel. The correct diagnosis was made at the time of operation. Cross-sectional imaging can be helpful in the evaluation of ventral hernias but was not diagnostic in this case. Regardless of the contents of a suspected strangulated hernia, operative intervention is required. Given the size of the defect after appendectomy and reduction of hernia, as and considering a potentially infected field, the decision was made to use a biologic mesh, with adherence to principles of hernia repair. A tension-free repair was undertaken with sufficient overlap of the fascial edges and mesh.

## Conclusions

Appendicitis containing umbilical hernia is a very rare phenomenon. Symptoms of an incarcerated or strangulated hernia along with CT imaging showing a blind-ended tubular structure in the hernia contents make appendicitis within an umbilical hernia likely. The principles of treatment of an incarcerated ventral hernia apply, as do the principles of management of acute appendicitis. Regardless of contents, the hernia contents must be fully explored and addressed at the time of operation and the defect must be closed appropriately.
